# Effects of clinical training on compliance with standard precautions among nursing students: a quasi-experimental study

**DOI:** 10.1186/s12909-026-09362-w

**Published:** 2026-05-04

**Authors:** Gul Hatice Tarakcioglu Celik, Sidika Kestel

**Affiliations:** Department of Fundamentals of Nursing, Faculty of Nursing, Hacettepe University, Ankara, Turkey

**Keywords:** Infection prevention and control, Standard precautions, Nursing education, Clinical training, Nursing student

## Abstract

**Background:**

Healthcare-associated infections are a safety concern that continues to impact the quality of patient care. Standard precautions have a structure that determines the steps and conceptual framework to be followed within the scope of infection prevention and control practices. The study aimed to evaluate the effect of the clinical training process on nursing students’ standard precautions compliance and attitudes.

**Methods:**

This study employed a quasi-experimental, single-group pre-test post-test design and was conducted at the Faculty of Nursing at a public university. 234 baccalaureate nursing students were included in the study. Before and after clinical training in the Spring Semester of the 2023–2024 Academic Year, the Compliance with Standard Precautions Scale was utilized to assess self-reported standard precautions compliance, while the Factors Influencing Adherence to Standard Precautions Scale-Student Version was used to evaluate attitudes toward standard precautions objectively.

**Results:**

Overall self-reported compliance increased from 69.04% to 72.96% after clinical training. In the FIASP-SV post-test, the mean “*justification*” score was significantly lower among 3rd -year students, whereas the mean “*practice culture*” score was significantly lower among 2nd -year students compared with the other class years. In the pre-test, a low-level positive correlation was observed between the total FIASP-SV and total CSPS scores (*r* = 0.164; *p* < 0.05).

**Conclusions:**

The findings of this study highlight the importance of designing a clinical training program that provides students with opportunities to experience nursing skills and procedures that require strict adherence to standard precautions. Nursing academics must address strategies in the nursing curriculum that will enable nursing students to transfer knowledge of infection prevention and control practices to clinical education.

## Background

Initially defined by the Centers for Disease Control (CDC) in 1987, standard precautions (SPs) were modified in 1996 to their current form. SPs provide guidance to protect individuals from blood, all body fluids and discharges, all materials contaminated with these fluids, regardless of whether bloody or not, and damaged skin and mucosa [1]. SPs for infection prevention and control (IPC) are an effective and primary means of protecting health-care workers (HCWs), patients, and their relatives/visitors [2]. SPs involve using specialized equipment and procedures to safeguard HCWs, such as nursing students, from exposure to infectious agents during clinical practice. For nursing students, SPs are crucial for IPC. This involves properly using personal protective equipment (PPE) such as gloves, masks, gowns, eye protection, and sometimes face shields. PPE serves as a barrier to reduce contact with infectious agents present in blood, bodily fluids, secretions, or contaminated surfaces. Besides PPE, SPs also includes practicing effective hand hygiene, safely disposing of contaminated materials, and keeping the environment clean [1, 2].

Adherence to the SPs reduces HAIs and the risk of cross-infection which can significantly impact the health and safety of both patients and HCWs [3]. For nursing students, learning and correctly implementing SP protocols are essential skills that support IPC and enhance personal protection during clinical practice. Mastering these practices ensures a safer healthcare environment and promotes the development of professional responsibility and competence in future nurses [4, 5].

In a review of literature evaluating nursing students’ SPs knowledge and practices and the teaching techniques used in education, about 35% of nursing students’ SPs knowledge was found to be at a moderate/good level, and their SPs practices and compliance were found to be at a moderate level [6]. Other studies found compliance to be less reassuring. A multicenter study conducted in Jordan found that only half of the students (51.3%) knew the SPs well, and 68.8% of the students’ practices were classified as “adequate.” The study reported a weak positive correlation between students’ knowledge and practice scores. This result supports the need for nursing educators to use different teaching methods and practices in effectively translating theoretical knowledge into safe practice [7]. In a study conducted in Nepal, student nurses had sufficient knowledge about SPs (medium-level knowledge = 91.3%), though their compliance rates were relatively low (65%). In line with these results, it was reported that students needed supportive supervision and feedback during the clinical practice process [8].

With increased experience or a more extended clinical practice period, students are expected to achieve better scores in knowledge, adaptation, and application processes. When the literature is evaluated, studies supporting this expectation are found. In a study conducted in Saudi Arabia, nearly half of the nursing students (45.83%) had acceptable general knowledge scores on IPC practices and SPs. A significant relationship was found between the students’ knowledge scores and the year they studied, with the highest scores being obtained by intern students (students in their last semester before graduation) with more extended clinical practice experience [9]. In another study emphasizing the positive effect of the clinical practice process on compliance with the SPs, nursing students’ knowledge and compliance with the SPs were found to be high, and a significant relationship was reported between the duration of practical training and compliance with the SPs (*p* = 0.01) [10]. In another study, the general compliance of student nurses to the SPs was found to be suboptimal (68.5%) due to poor compliance with proper hand washing and use of PPE, with “*leadership*” and “*contextual cues*” identified as the main factors affecting self-reported compliance. These findings again emphasize the need for nursing students to receive more intensive theoretical and practical training in IPC and opportunities to develop leadership and interdisciplinary communication skills before clinical practice [11].

In Türkiye, recent catastrophic events such as the COVID-19 pandemic and the 2023 earthquakes disrupted nursing education, forcing temporary shifts to online and hybrid learning for field courses. These interruptions may have influenced students’ opportunities to develop psychomotor and infection-control skills, underscoring the need to examine how clinical training affects their compliance with SPs.

To determine if nursing students are developing the skills and attitudes needed to protect themselves from exposure to infection, this study evaluated the effect of the clinical practice process on nursing students’ SPs compliance and attitudes.

## Method

### Study design and sample

In this quasi-experimental study with a single-group and pre-test/post-test design, we evaluated how clinical practice affected student nurses’ compliance and attitudes toward SPs. A self-reported survey was completed by the 2nd -to-4th -year nursing students who had completed the fundamentals of nursing course. The target population was 594 nursing students, including 187 in the 2nd -year, 197 in the 3rd -year, and 210 in the 4th -year. The desired sample size was determined as minimum 234 subjects, with a 95% power and a 0.05 alpha level, using the sampling of the known population formula. The sample was stratified to represent the population from each year, and the study was finally completed with 82 (35.0%) students for the 2nd -year, 72 (30.8%) students for the 3rd -year, and 80 (34.2%) students for the 4th -year to achieve proportionally adequate representation from each year.

### Theoretical framework

In nursing education, the classroom environment and the clinical environment are interconnected. Students must apply what they learn in the classroom in the clinic. Because nursing is a professional practice discipline, the performance of nursing students in clinical practice is more important than what they can show in the classroom. While clinical practice allows students to experience their theoretical knowledge in a real environment and realize themselves, classroom teaching prepares students for the clinical environment. However, expecting students to continue learning in clinical education is unnecessary. In clinical practice, students put their knowledge into practice, learn to solve problems, think critically, make decisions, and use clinical reasoning [12].

Many theories and models are used in the realization of learning. In one of these, Experiential Learning Theory, Kolb defines learning as “*knowledge is formed through the transformation of experiences*.” Kolb’s theory includes a cyclical process consisting of four stages: *Concrete Experience*, which includes learning by “*feeling/touching*”; *Reflection Observation*, which provides for learning by “*watching”*; *Abstract Conceptualization*, which includes learning by “*thinking*”; and *Active Experimentation*, which includes learning by “*doing*.” While students are allowed to have concrete experiences during clinical practice, opportunities are provided for them to live these experiences repeatedly, and the steps of reflection, thinking, and taking action are attempted to be completed in the realization of learning [13]. While nursing students learn based on practice, they frequently use the “*Active Experimentation*” stage, which includes learning by doing, in transforming theoretical knowledge into practice [14]. The effect of clinical practice on evaluating compliance with and attitude toward SPs was designed in line with Kolb’s theory, as students transformed their experiences into concrete learning, repeated these actions, and performed the steps of thinking, reflecting, and acting [13]. In summary, the reflection of the relationship between theory and practice on compliance and attitude towards SPs was evaluated.

### Survey instruments

The survey instrument comprised three parts: student demographic items, the Compliance with Standard Precautions Scale (CSPS), and the Factors Influencing Adherence to Standard Precautions Scale-Student Version (FIASP-SV) scale.

The CSPS was developed by Lam [15] to evaluate compliance with SPs, and Samur et al. [16] adapted this scale for use in Turkish. It has good psychometric properties, with responses rated using a 4-point Likert format (“never”, “seldom”, “sometimes”, and “always”). The scale consists of 20 items, and items 2, 4, 6, and 15 were reverse-scored. In reverse-scored items, one point is given to the answer “*never*”; in positive items, one point is given to the answer “*always*”; the scale is scored by giving zero points to the other answers. The minimum score from the scale is 0, and the maximum score is 20. The five dimensions of CSPS are: “*use of protective equipment/devices*,” “*disposal of sharps*,” “*disposal of waste*,*”* “*decontamination of spills and used equipment or articles*,*”* and “*prevention of cross infection from person to person*.” The original scale had an internal consistency coefficient of 0.73, while the adapted version was 0.71 [15, 16]. In the present study, the scale showed acceptable internal consistency with a Cronbach’s alpha of 0.732.

FIASP-SV was designed by Bouchoucha et al., [17] to assess attitudinal and workplace factors influencing HCWs’ adherence to SPs. Örücü & Atay [18] validated the Turkish version of the FIASP-SV with nursing students. The scale consists of 23 items and four sub-dimensions, rated on a 5-point Likert-type scale (from 0 = not all to 4 = very much). The original version’s Cronbach’s alpha values were 0.75 for leadership, 0.80 for justification, 0.66 for practice culture, and 0.79 for contextual cues [17]. The adapted Turkish version yielded a total Cronbach’s alpha value was 0.623 (*leadership* = 0.810, *justification* = 0.818, *practice culture* = 0.585, and *contextual cues* = 0.847) [18]. In the present study, the scale demonstrated acceptable internal consistency (total = 0.711, *leadership* = 0.815, *justification* = 0.780, *practice culture* = 0.684, and *contextual cues* = 0.716).

Written permission was obtained from the authors who conducted the Turkish validity and reliability study of the scales used in the study.

### Data collection

Eligible students were invited to take the survey by visiting them during the hours of the relevant field courses before they started their clinical/field practices during the Spring Semester of the 2023–2024 Academic Year. Written informed consent was obtained from the students who voluntarily agreed to participate in the study. Then, participating students completed the survey twice (pre-/post-test), one week before starting the clinical practices and again during the last week of the clinical practices. To enable paired comparison while preserving anonymity, students were asked to use the same self-generated nickname on both the pre-test and post-test forms. Surveys were matched only when the same nickname appeared on both forms; unmatched forms were excluded from the paired analyses.

### Data analysis

The study data were evaluated using the IBM SPSS Statistics 26.0 (IBM, Armonk, NY, USA) program. Frequency distributions were used for categorical variables, and descriptive statistics (mean, standard deviation, median) were used for numerical variables. The reliability of the scales used in the study was examined using Cronbach’s Alpha internal consistency coefficient.

A dependent sample t-test was used to examine whether there was a difference between the scales’ pre-test and post-test scores. Pearson’s correlation coefficient was used to determine the relationship between FIASPS and CSPS sub-domains. One-way analysis of variance (ANOVA) was used to compare class years, and an independent samples t-test was used to compare IPC training in FIASPS and CSPS.

### Ethical approval

The ethics committee approval for the study was obtained from the Hacettepe University Social and Human Sciences Ethics Committee (Date: October 14, 2023, ID: E-66777842-300-00003204319). After the necessary ethics committee approval was obtained, an implementation permission letter was obtained from the Hacettepe University Faculty of Nursing, where the study would be conducted. The student nurses who voluntarily participated were included in the study after their written informed consent was obtained.

## Results

### Demographic characteristics

The demographic characteristics of the study participants (*n* = 234) are presented in Table 1. Most of nursing students were female (88.0%), with an average age of 22.28 ± 1.392. They received IPC training both before and after clinical practices (87.2% and 92.3%, respectively). 85.9% of the students stated that they had previously heard of the concept of SPs.

**Table 1:** Demographic characteristics of students

Demographic Characteristics	(X̄±SD)	
Age	22.28 ± 1.392	
	*n* = 234	%
Year		
2nd	82	35.0
3rd	72	30.8
4th	80	34.2
Gender		
Female	206	88.0
Male	28	12.0
Receiving IPC training before clinical practice		
Yes	204	87.2
No	30	12.8
Receiving IPC training after clinical practice		
Yes	216	92.3
No	18	7.7
Heard of the concept of SPs		
Yes	201	85.9
No	33	14.1

*IPC* Infection prevention and control, *SD* Standard deviation, *SPs* Standard precautions, X̄: Mean

### Compliance with SPs

Table 2 shows participants’ self-reported compliance at the pre-test and post-test on the five key domains of SPs practice. The overall (69.04% and 72.96%, respectively) and domain-specific compliance rates (from 59.6% to 76.2% to %59.9–79.0%, respectively) improved in the post-test compared to the pre-test. After the post-test, self-reported compliance decreased in four items: “*I change gloves between patient contacts* (item 11: from 81.2% to 72.7%), *“I reuse a surgical mask or disposable Personal Protective Equipment (PPE)* (item 15: from 64.1% to 60.7%)”, “*I recap used needles after giving an injection* (item 4: from 65.4% to 64.6%)”, and “*I only use water for hand washing* (item 2: from 56.8% to 54.3%)”. The lowest (item 3: 26.1%, %29.5, respectively) and highest (item 5: %96.6 for each) self-reported compliance items were the same for the pre-test and post-test (Table 2).

**Table 2:** Nursing students’ self-report of SPs compliance

CSPS Items	Current study (*n* = 234)									
	Never (%)		Seldom (%)		Sometimes (%)		Always (%)		Compliance Rate (%)	
	Pre-test	Post-test	Pre-test	Post-test	Pre-test	Post-test	Pre-test	Post-test	Pre-test	Post-test
*Use of protective equipment/devices*										
7. I remove Personal Protective Equipment (PPE) in a designated area.	4.7	2.6	9.0	5.1	24.3	23.1	62.0	69.2	62.0	69.2
10. I wear gloves when I am exposed to body fluids, blood products and any excretion of patients.	-	-	0.4	0.9	5.6	4.7	94.0	94.4	94.0	94.4
13. I wear a surgical mask alone or in combination with goggles, face shield and apron whenever there is a possibility of a splash or splatter.	2.6	1.3	16.2	13.2	32.5	33.3	48.7	52.2	48.7	52.2
14. My mouth and nose are covered when I wear a mask.	0.9	-	0.4	0.9	6.8	4.7	91.9	94.4	91.9	94.4
15. I reuse a surgical mask or disposable Personal Protective Equipment (PPE).	64.1	60.7	22.6	18.8	7.3	11.5	6.0	9.0	64.1	60.7
16. I wear a gown or apron when exposed to blood, body fluids or any patient excretions.	4.3	3	14.5	10.3	29.1	29.9	52.1	56.8	52.1	56.8
*Average Overall Compliance*									*68.8*	*71.2*
*Disposal of sharps*										
4. I recap used needles after giving an injection.	65.4	64.6	17.1	17.9	6.4	6.4	11.1	11.1	65.4	64.6
5. I put used sharp articles into sharps container.	1.3	2.1	0.4	-	1.7	1.3	96.6	96.6	96.6	96.6
6. The sharps container is disposed of when its contents reach the full line on the container.	66.7	69.7	14.5	16.7	12.2	8.0	6.8	5.6	66.7	69.7
*Average Overall Compliance*									*76.2*	*76.9*
*Disposal of waste*										
17. Waste contaminated with blood, body fluids, secretion and excretion is placed in red plastic bags irrespective of the patient’s infection status.	5.6	7.3	7.7	3.0	16.7	10.7	70.0	79.0	70.0	79.0
*Average Overall Compliance*									*70.0*	*79.0*
*Decontamination of spills and used equipment or articles*										
8. I take a shower in case of extensive splashing even after I have put on Personal Protective Equipment (PPE)	2.1	2.1	9.4	5.6	27.8	26.5	60.7	65.8	60.7	65.8
18. I decontaminate surfaces and equipment after use.	2.1	-	4.3	1.7	20.9	14.5	72.6	83.8	72.6	83.8
19. I wear gloves to decontaminate used equipment with visible soils.	0.4	-	2.6	1.3	15.0	12.4	82.0	86.3	82.0	86.3
20. I clean up spillage of blood or other body fluids immediately with disinfectants.	5.6	1.8	7.7	5.1	19.7	17.5	67.1	75.6	67.1	75.6
*Average Overall Compliance*									*70.6*	*77.8*
*Prevention of cross infection from person-to-person*										
1. I wash my hands between patient contacts.	-	-	5.1	4.7	27.8	28.2	67.1	67.1	67.1	67.1
2. I only use water for hand washing.	56.8	54.3	29.5	31.6	11.1	10.3	2.6	3.8	56.8	54.3
3. I use alcoholic hand rubs as an alternative to soap and water if my hands are not visibly soiled.	4.3	3.0	17.1	15.8	52.6	51.7	26.1	29.5	26.1	29.5
9. I would cover my wound(s) or lesion(s) with waterproof dressing before patient contacts.	6.0	3.8	17.5	10.7	29.9	30.3	46.6	55.2	46.6	55.2
11. I change gloves between patient contacts.	0.4	-	1.7	3.8	16.7	23.5	81.2	72.7	81.2	72.7
12. I decontaminate my hands immediately after removal of gloves.	-	-	2.6	1.7	17.1	17.5	80.3	80.8	80.3	80.8
*Average Overall Compliance*									*59.6*	*59.9*
Average overall compliance for the Total CSPS									69.04	72.96

*CSPS* Compliance with Standard Precautions Scale

### Scales’ comparison results with demographic data

In the CSPS pre-test, there were significant differences in the sub-dimension of “*use of protective equipment/devices*” (2nd and 3rd -years compared to 4th -year, p = 0.030), “*disposal of waste*” (2nd -year compared to 3rd -4th -years, p = 0.007), and “*the prevention of cross infection from person-to-person*” (2nd -year compared to 4th -year, p = 0.041). The difference in the sub-dimension of the “*disposal of sharps”* between the mean scores of the students who received and did not receive training before clinical practice on IPC is statistically significant (p = 0.001) (Table 3).

**Table 3:** Examining the differences between demographic characteristics according to scales and sub-dimension scores (pre-test)

		Compliance with standard precautions scale						Factors influencing adherence to standard precautions scale-student version				
		Total	Use of protective equipment / devices	Disposal of sharps	Disposal of waste	Decontamination of spills and used equipment or articles	Prevention of cross infection from person-to-person	Total	Leadership	Justification	Practice culture	Contextual cues
	*n*	X̄±SD	X̄±SD	X̄±SD	X̄±SD	X̄±SD	X̄±SD	X̄±SD	X̄±SD	X̄±SD	X̄±SD	X̄±SD
Class												
2nd year	82	13.65 ± 3.743	4.91 ± 1.650	2.21 ± 0.857	0.57 ± 0.498	2.10 ± 1.001	3.85 ± 1.167	3.35 ± 0.367	4.05 ± 0.657	1.76 ± 0.807	3.32 ± 0.893	4.35 ± 0.620
3rd year	72	14.04 ± 2.765	4.94 ± 1.197	2.38 ± 0.740	0.78 ± 0.419	2.43 ± 0.869	3.51 ± 1.343	3.33 ± 0.310	4.01 ± 0.729	1.59 ± 0.648	3.24 ± 0.945	4.53 ± 0.514
4th year	80	12.93 ± 3.438	4.36 ± 1.686	2.29 ± 0.732	0.76 ± 0.428	2.15 ± 1.008	3.36 ± 1.265	3.29 ± 0.291	3.88 ± 0.679	1.71 ± 0.696	3.31 ± 0.821	4.36 ± 0.491
F; p		2.177; 0.116	3.572; 0.030^*^	0.885; 0.414	5.080; 0.007^**^	2.584; 0.078	3.243; 0.041^*^	0.675; 0.510	1.324; 0.268	1.324; 0.268	1.172; 0.312	0.209; 0.811
Difference***		-	2.3-4	-	2-3.4	-	2–4	-	-	-	-	-
Receiving infection control and prevention training												
Yes	204	13.51 ± 3.349	4.69 ± 1.550	2.35 ± 0.750	0.71 ± 0.455	2.21 ± 0.977	3.56 ± 1.272	3.32 ± 0.315	3.97 ± 0.690	1.67 ± 0.705	3.26 ± 0.885	4.42 ± 0.509
No	30	13.57 ± 3.636	5.07 ± 1.574	1.87 ± 0.860	0.63 ± 0.490	2.27 ± 0.944	3.73 ± 1.258	3.37 ± 0.386	4.02 ± 0.685	1.80 ± 0.848	3.51 ± 0.848	4.33 ± 0.778
t; p		-0.078; 0.938	-1.252; 0.212	3.218; 0.001^**^	0.863; 0.389	-0.294; 0.769	-0.703; 0.483	-0.898; 0.370	-0.371; 0.711	-0.926; 0.355	-1.460; 0.146	0.882; 0.379

F: One-way Analysis of Variance (ANOVA), *SD* Standard deviation, t: Independent Samples T Test, X̄: Mean

*p < 0.05, **p < 0.01, *** Tukey Test

In the CSPS post-test, total score and the sub-dimensions of “*use of protective equipment/devices*”, “*decontamination of spills and used equipment or articles”*, and “*prevention of cross infection from person-to-person*” scores of the 3rd -year students were significantly higher than the 2nd and 4th -year students (p = 0.009, p = 0.021, p = 0.049, p = 0.003, respectively) (Table 4).

**Table 4:** Examining the differences between demographic characteristics according to scales and sub-dimension scores (post-test)

		Compliance with standard precautions scale						Factors influencing adherence to standard precautions scale-student version				
		Total	Use of protective equipment / devices	Disposal of sharps	Disposal of waste	Decontamination of spills and used equipment or articles	Prevention of cross infection from person-to-person	Total	Leadership	Justification	Practice culture	Contextual cues
	*n*	X̄±SD	X̄±SD	X̄±SD	X̄±SD	X̄±SD	X̄±SD	X̄±SD	X̄±SD	X̄±SD	X̄±SD	X̄±SD
Class												
2nd class	82	13.77 ± 3.598	4.80 ± 1.659	2.38 ± 0.780	0.76 ± 0.432	2.35 ± 0.921	3.48 ± 1.533	3.41 ± 0.304	4.11 ± 0.585	1.96 ± 0.829	2.98 ± 0.968	4.43 ± 0.599
3rd class	72	15.11 ± 3.133	5.36 ± 1.367	2.24 ± 0.741	0.81 ± 0.399	2.65 ± 0.632	4.06 ± 1.221	3.30 ± 0.343	4.03 ± 0.716	1.56 ± 0.654	3.47 ± 1.021	4.36 ± 0.599
4th class	80	13.49 ± 3.435	4.69 ± 1.643	2.30 ± 0.701	0.81 ± 0.393	2.39 ± 0.834	3.30 ± 1.409	3.38 ± 0.300	3.99 ± 0.612	1.93 ± 0.694	3.34 ± 0.925	4.33 ± 0.532
F; p		4.857; 0.009^**^	3.932; 0.021^*^	0.709; 0.493	0.455; 0.635	3.055; 0.049^*^	5.967; 0.003^**^	2.078; 0.128	0.734; 0.481	6.971; 0.001^*^	5.428; 0.005^**^	0.766; 0.466
Difference***		3-2.4	3-2.4	-	-	3-2.4	3-2.4	-	-	3-2.4	2-3.4	-
Receiving infection control and prevention training												
Yes	216	14.06 ± 3.484	4.92 ± 1.615	2.32 ± 0.738	0.80 ± 0.400	2.46 ± 0.817	3.56 ± 1.439	3.36 ± 0.318	4.04 ± 0.639	1.81 ± 0.721	3.31 ± 0.944	4.35 ± 0.585
No	18	14.44 ± 3.240	5.17 ± 1.249	2.17 ± 0.786	0.67 ± 0.485	2.44 ± 0.856	4.00 ± 1.283	3.40 ± 0.299	4.02 ± 0.618	1.98 ± 1.063	2.57 ± 1.257	4.64 ± 0.377
t; p		-0.457; 0.648	-0.641; 0.522	0.840; 0.402	1.142; 0.268	0.069; 0.945	-1.255; 0.211	-0.455; 0.649	0.163; 0.871	-0.875; 0.382	2.434; 0.025^*^	-2.087; 0.038^*^

F: One-way Analysis of Variance (ANOVA), *SD* Standard deviation, t: Independent Samples T Test, X̄: Mean

*p < 0.05, **p < 0.01, *** Tukey Test

In the FIASP-SV post-test, the mean “*justification*” score was significantly lower among 3rd -year students, whereas the mean “*practice culture*” score was significantly lower among 2nd -year students compared with students in the other class years (*p* = 0.001, *p* = 0.005, respectively). The difference in the sub-dimensions of the “*practice culture*” and “*contextual cues*” between the mean scores of the students who received and did not receive IPC training was statistically significant (*p* = 0.025 and *p* = 0.038, respectively) (Table 4).

### Pre-test-post-test comparison results of scales and correlation analysis

There was a statistically significant difference between the mean pre-test and post-test total CSPS scores (*p* = 0.041). In addition, statistically significant differences were found in the CSPS sub-dimensions of “*disposal of waste*” and “*decontamination of spills and used equipment or articles*” (*p* = 0.018, *p* = 0.002, respectively). In the FIASP-SV, a statistically significant difference between the pre-test and post-test mean scores was found only in the “*justification*” sub-dimension (*p* = 0.019) (Table 5).

**Table 5:** Pre-test and post-test comparison results of scales

	Pre-Test	Post-Test	
	X̄±SD	X̄±SD	t; *p*
CSPS	13.52 ± 3.379	14.09 ± 3.461	-2.058; 0.041^*^
*Sub-Dimensions*			
Use of protective equipment/devices	4.74 ± 1.555	4.94 ± 1.589	-1.495; 0.136
Disposal of sharps	2.29 ± 0.78	2.31 ± 0.741	-0.369; 0.713
Disposal of waste	0.70 ± 0.459	0.79 ± 0.408	-2.386; 0.018^*^
Decontamination of spills and used equipment or articles	2.22 ± 0.971	2.46 ± 0.818	-3.191; 0.002^**^
Prevention of cross infection from person-to-person	3.58 ± 1.269	3.59 ± 1.430	-0.111; 0.911
FIASP-SV	3.32 ± 0.325	3.37 ± 0.316	-1.550; 0.123
*Sub-Dimensions*			
Leadership	3.98 ± 0.688	4.04 ± 0.636	-1.324; 0.187
Justification	1.69 ± 0.724	1.83 ± 0.751	-2.369; 0.019^*^
Practice Culture	3.29 ± 0.883	3.25 ± 0.988	0.472; 0.637
Contextual Cues	4.41 ± 0.550	4.37 ± 0.576	0.791; 0.430

*SD* Standard deviation, t: Dependent Samples T Test, X̄: Mean

*p < 0.05, **p < 0.01

The Pearson Correlation Coefficient was used to analyze the relationship among sub-dimensions of the FIASP-SV and the CSPS. Among the pre-test scores, a low-level positive and statistically significant correlation was found between the total FIASP-SV and the CSPS scores (*r* = 0.164; *p* < 0.05) (Table 6).

**Table 6:** Examining the relationships between pre-test/post-test scale scores

Pre-test						
		FIASP-SV	Leadership	Justification	Practice Culture	Contextual Cues
CSPS	r	0.164	0.404	-0.310	0.092	0.229
	p	0.012 ^*^	0.000^***^	0.000^***^	0.162	0.000^***^
Use of protective equipment/devices	r	0.148	0.359	-0.242	0.096	0.154
	p	0.024^*^	0.000^***^	0.000^***^	0.142	0.018^*^
Disposal of sharps	r	0.070	0.133	-0.156	0.026	0.179
	p	0.287	0.043^*^	0.017^*^	0.697	0.006^**^
Disposal of waste	r	-0.017	0.134	-0.212	0.026	0.082
	p	0.794	0.041^*^	0.001^*^	0.694	0.210
Decontamination of spills and used equipment or articles	r	0.112	0.265	-0.232	0.002	0.238
	p	0.087	0.000^***^	0.000^***^	0.973	0.000^***^
Prevention of cross infection from person-to-person	r	0.132	0.304	-0.179	0.099	0.099
	p	0.043^*^	0.000^***^	0.006^**^	0.129	0.130

Post-test						
		FIASP-SV	Leadership	Justification	Practice Culture	Contextual Cues
CSPS	r	0.016	0.328	-0.444	0.295	0.079
	p	0.812	0.000^***^	0.000^***^	0.000^***^	0.227
Use of protective equipment/devices	r	0.020	0.285	-0.345	0.279	0.011
	p	0.764	0.000^***^	0.000^***^	0.000^***^	0.870
Disposal of sharps	r	-0.069	0.104	-0.236	0.100	0.010
	p	0.292	0.111	0.000^***^	0.129	0.873
Disposal of waste	r	-0.001	0.106	-0.163	0.037	0.083
	p	0.988	0.107	0.013^*^	0.571	0.207
Decontamination of spills and used equipment or articles	r	0.029	0.158	-0.276	0.152	0.151
	p	0.656	0.016^*^	0.000^***^	0.020^*^	0.021^*^
Prevention of cross infection from person-to-person	r	0.035	0.303	-0.365	0.255	0.064
	p	0.592	0.000^***^	0.000^***^	0.000^***^	0.326

r: Pearson Correlation Coefficient

*p < 0.05, **p < 0.01

## Dıscussıon

This study explored 2nd -to-4th -year nursing students’ compliance with SPs and factors influencing adherence to SPs in Türkiye. Based on student nurses’ self-reports, compliance with SPs was above average before and after clinical practice (pre-/post-test), consistent with previous studies [15, 19]. These findings should be interpreted in the context of the educational disruptions experienced in recent years, including the COVID-19 pandemic and the 2023 earthquakes, which may have influenced students’ prior opportunities to develop clinical and IPC skills.

In addition, the participants reported above-average compliance with SPs before and after clinical training (69.04%, 72.96%, respectively). Although the overall compliance is consistent with the literature [4, 11, 17, 20], no change in some items (item 1: washing hands between patient, and item 5: put used sharp articles into sharps container), or decreased compliance in reverse-scored items of 2 (only use water for hand washing), 4 (recap used needles after giving an injection), and 15 (reuse a surgical mask or disposable PPE) and item of 11 (change gloves between patient contacts) after clinical training should be considered with caution (Table 2). One of the first requirements is ensuring that the nursing curriculum structure ensures students have up-to-date IPC practices [21]. These findings highlight the importance of developing strategies to promote appropriate use of PPE by all HCWs, adherence to waste disposal standards, and compliance with IPC practices to prevent cross-infections [22]. Promoting a culture in which compliance with IPC is prioritized in the clinical setting and having appropriate role models will effectively increase compliance and awareness among nursing students.

According to both pre-test and post-test analysis results, nursing students showed the least compliance in the “*prevention of cross infection from person-to-person*” sub-dimension (59.6%, 59.9%, respectively) (Table 2). The compliance rates of some of the items in this section are pretty significant. Correct and effective hand hygiene is an extremely important strategy in IPC practices [23]. However, the sample showed the lowest compliance with one of the hand hygiene items of, “*I use alcoholic hand rubs as an alternative to soap and water if my hands are not visibly soiled*” both before and after the clinic (26.1%, 29.5%, respectively) (Table 2). Likewise, students who self-reported that they performed hand hygiene using only water, although it was not appropriate as stated in the guidelines [2, 24], constituted half of the sample (56.8%, 54.3%, respectively), while the proportion of students who declared that they changed their gloves between patients decreased by about 10% in the post-test (Table 2). The nursing curriculum should prepare student nurses to provide effective and efficient patient care [25]. The clinical setting should support a culture in which strict adherence to IPC practices is paramount [20]. Thus, student nurses can transfer their theoretical knowledge to practice. In a qualitative study, nursing students shared their experiences of IPC practices and discussed the difficulties of compliance with SPs when paired with noncompliant nurses [26]. Inadequate knowledge of SPs is a concern for faculty, and the findings of this study can be used as an informative example for nursing students and nurses because hospital culture affects practice regardless of knowledge.

In pre-test 2nd -year students more likely to comply on the sub-dimensions of “*use of protective equipment / devices*,” “*disposal of waste*,” and “*prevention of cross infection from person-to-person*” than the 3rd -year and/or 4th -year students (Table 3). A possible reason for these differences may be that 4th -year and 3rd -year students did not have sufficient clinical practice experience during the COVID-19 pandemic and after the two major earthquakes in the country. Although attempts were made to complete the deficit in education with different alternative education models for IPC practices known to be effective during this process [27], the effects of completing the nursing fundamentals course with distance education were seen again in this study [28].

After clinical training in the post-test 3rd -year students were more likely to comply on the sub-dimensions of “use of protective equipment/devices,” “decontamination of spills and used equipment or articles,” and “prevention of cross infection from person-to-person” than 2nd -year and 4th -year students (Table 4). The fact that all 3rd -year students perform their clinical practice in specialized units in the field of pediatrics, that in-service training provided to students in these clinics is relatively more, and that being in a specialized field as a driving force for being more careful, may be among the possible reasons for these results [29].

In the FIASP-SV, there were significant differences only post-test. The “*justification*” score average of the 3rd -year students was significantly lower than the other classes (Table 4). The justification factor consists of items in which participants provide reasons for not complying with the SPs in IPC practices, and a low average score in this factor is a positive result [30]. The fact that 3rd -year students had a lower average than the justification factor in the evaluation made after clinical practice can be explained by the fact that the hospitals and clinics where the practice was conducted were pediatrics, considered a specialized field. Also, 3rd -year students are exposed to more clinical stimuli than other years, such as in-service training, clinical team leaders, and even first-degree relatives (primarily parents) who are responsible for the care of patients. These findings support the proposition that well-executed clinical leadership can override justification [17, 31]. However, nursing educators face a challenge in encouraging student nurses to appreciate the risks to themselves and their patients if they fail to follow SPs guidelines in IPC practice [11].

Because 2nd -year students have less clinical experience than in other years may explain their lower scores on the factor “*practice culture*.” Another unsurprising result after the post-test was that student nurses who reported receiving training in IPC had significantly higher mean scores on the “*practice culture*” and “*contextual clues factors*” (Table 4). The “*practice culture*” factor addresses professional practices that encourage or discourage using SPs in institutions [30]. Institutions’ IPC climates must encourage staff to comply with SPs [32, 33]. In line with the literature [20, 21], nursing students have applied clues in putting theoretical concepts of SPs into practice with IPC training they receive in addition to their curriculum. Thus, using habits correctly in the clinical environment can be an effective strategy in encouraging students to comply with SPs in the future [34].

In the pre-test/post-test comparison, the total CSPS score and CSPS sub-dimensions of “*disposal of waste*” and “*decontamination of spills and used equipment or articles*” were significantly higher in the post-test than in the pre-test (Table 5). In contrast, no statistically significant difference was observed in the total FIASP-SV score, although a significant difference was found in the “*justification*” sub-dimension (Table 5). Because lower scores in the “justification” sub-dimension indicate fewer perceived reasons for not adhering to SPs, this finding may be interpreted as favorable. Taken together, these findings suggest that clinical training may have supported improvements in selected aspects of self-reported compliance and in one attitude-related dimension, although the overall change in attitudes was limited [17].

A low-level positive correlation was observed between the total FIASP-SV and the total CSPS pre-test scores (Table 6), suggesting that more favorable attitudes toward SPs may be associated with higher self-reported compliance before clinical training. These findings highlight the importance of creating educational opportunities that explicitly link clinical practice with IPC knowledge and support students in translating theoretical knowledge into practice [35, 36]. Nursing faculty should design clinical training programs that provide students with opportunities to experience nursing skills and procedures that require strict adherence to SPs [4]. Such efforts may contribute to improving the quality of care and protecting students’ health [37].

### Limitations

This study has several limitations that should be considered when interpreting the findings. First, the study used a quasi-experimental single-group pre-test/post-test design without a control group; therefore, changes observed between the pre-test and post-test should not be interpreted as definitive causal effects of clinical training alone. Second, participation was voluntary, which may have introduced selection bias. Third, approximately 80 survey forms could not be included in the paired analyses because the pre-test and post-test forms could not be matched using the self-generated nicknames, which may have reduced the representativeness of the final analytic sample. Fourth, compliance with SPs was assessed using self-report measures, which may be subject to recall and social desirability bias. Finally, the study was conducted in a single nursing faculty, which may limit the generalizability of the findings to other nursing schools and clinical training settings in Türkiye. In addition, broader educational disruptions related to the COVID-19 pandemic and the 2023 earthquakes may have influenced students’ prior clinical exposure and perceptions of risk, although the extent of this effect could not be directly measured in the present study.

## Conclusion

This study suggests that clinical training may contribute to improvements in nursing students’ self-reported compliance with SPs and may help shape attitudes related to IPC practices. However, despite above-average overall compliance scores, important gaps remained in several critical practices, particularly those related to hand hygiene and prevention of cross-infection. These findings indicate that clinical exposure alone may not be sufficient to ensure consistent adherence to SPs.

The findings underscore the importance of strengthening undergraduate nursing curricula and clinical learning environments to better support the transfer of IPC knowledge into safe clinical practice. In particular, nursing faculty and clinical educators should provide students with structured opportunities to practice skills requiring strict adherence to SPs, while also promoting positive role modeling and a supportive clinical culture. Future studies using multi-center designs and controlled comparisons are needed to better clarify the specific contribution of clinical training to students’ compliance with SPs.

## Data Availability

The datasets generated and/or analyzed during the current study are not publicly available but are available from the corresponding author on reasonable request.

## Abbreviations

CDC: Centers for Disease Control
CSPS: Compliance with Standard Precautions Scale
FIASP-SV: Factors Influencing Adherence to Standard Precautions Scale-Student Version
HAIs: Healthcare-associated infections
HCWs: Health-care workers
IPC: Infection prevention and control
PPE: Personal protective equipment
SPs: Standard precautions
